# Supplementary material for: A Systematic Review and Meta-Analysis of the Incidence and Risk Factors for Major Adverse Cardiovascular Events in Patients with Unrepaired Abdominal Aortic Aneurysms
Source: Biomedicines. 2023 Apr 14;11(4):1178. doi: 10.3390/biomedicines11041178 (PMC10135825; doi:10.3390/biomedicines11041178)
Supplement: Supplementary file 1 [file biomedicines-11-01178-s001.zip › biomedicines-2278545-supplementary.pdf]

Search strategy

Databases: Medline (Ovid), Scopus

Search string:

"abdominal aortic aneurysm" OR "abdominal aortic aneurysms" OR “AAA”

AND

((“cause of death” OR “survival rate” OR “disease progression” OR “prognosis” OR “severity of illness index” OR “risk factors”

AND

“cohort studies”)

OR

“myocardial ischaemia” OR “myocardial infarction” OR “stroke” OR “cerebrovascular disorders”)

Supplementary Table S1: Quality assessment questionnaire to determine the risk of bias of the included studies

Quality category	Explanatory Criteria	Response		
		Yes	Partial	No
Objective defined	Study objective defined in the introduction section as examining the association of AAA with MACE and/ or clinically-relevant events	2		
	Study objective incompletely reported; substantial information has to be gathered from parts of the paper other than the introduction/background/objective section		1	
	Question or objective is not reported, or is incomprehensible.			0
Study design described	Study is defined as prospective design	2		
	Study designed is retrospective		1	
	Study design not reported			0
Sample size estimation	Sample size estimation performed and methodology for sample size estimation clearly explained.	2		
	Samples size estimation performed but the study did not achieve the required sample size, and the reason is explained.		1	
	No or limited sample size estimation reported			0
Unrepaired AAA	Patients with unrepaired AAA reported.	2		
	AAA repair status not reported			0
Reporting of Major Adverse Cardiovascular Events	All of MI, stroke and cardiovascular death reported	2		
	Only 1-2 of MI, non-fatal stroke and cardiovascular death reported		1	
	No mention of stroke or MI			0
Assessment of primary outcome	Independent blind assessment of cardiovascular death	2		
	Confirmation of cardiovascular death with death certificate or medical records		1	
	Determination of cardiovascular death by third party report or not reported			0
		2		

Participant characteristics described	At least 3 of the following characteristics of cohort described: Age, sex, hypertension, diabetes, coronary heart disease, body mass index and smoking history.			
	Only two of following participant characteristics described: Age, sex, hypertension, diabetes, coronary heart disease, body mass index and smoking history.		1	
	No participant characteristics reported			0
Duration of follow up	Cohort was followed up over >3 years	2		
	Cohort was followed up for 1-3 years		1	
	Cohort was followed up for <1 year			0
Statistical methods	All statistical methods used to control for confounding were described and age, sex, hypertension, diabetes, coronary heart disease and smoking adjusted for	2		
	Statistical methods used to control for confounding were partially described or incomplete adjustment for confounders		1	
	Statistical methods used to control for confounding not described or no adjustment for confounders			0
